# Genetic barcodes allow traceability of CRISPR/Cas9-derived* Aspergillus niger* strains without affecting their fitness

**DOI:** 10.1007/s00294-021-01164-5

**Published:** 2021-03-16

**Authors:** Sandra Garrigues, Roland S. Kun, Ronald P. de Vries

**Affiliations:** Fungal Physiology, Westerdijk Fungal Biodiversity Institute & Fungal Molecular Physiology, Utrecht University, Uppsalalaan 8, 3584 CT Utrecht, The Netherlands

**Keywords:** CRISPR/Cas9, *Aspergillus niger*, Traceability, Barcoding, Risk assessment, Transcription factors

## Abstract

Safe use of genetically modified organisms (GMOs) in biotechnology requires the ability to track the presence of these strains in any environment in which they are applied. For this, introduction of genetic barcodes within the editing site represents a valuable tool for the identification of microbial strains that have undergone genetic modifications. However, it is not known whether these barcodes would have any unexpected effect in the resulting strains or affect the efficiency of the genetic modification. CRISPR/Cas9 has become one of the fastest-growing technologies for genome editing in a range of organisms, including fungi. However, this technology enables the generation of scarless GMOs that are very difficult to distinguish from naturally occurring mutants or other modified organisms. In this study, we address this issue using the industrial workhorse *Aspergillus niger* as a test case. We applied CRISPR/Cas9 technology to delete the genes encoding the transcriptional regulators XlnR and AraR, involved in the production of plant biomass-degrading enzymes. We generated 20-bp barcoded and non-barcoded Δ*xlnR* and Δ*araR* mutants and analyzed the traceability and fitness of the resulting strains, as well as the efficiency of the genetic modification. Results showed that both barcoded and non-barcoded mutants can be traced by routine PCR reactions when the specific CRISPR/Cas9 modification is known. Additionally, barcodes neither affected the efficiency of the genetic modification nor the growth or protein production of the resulting strains. These results confirm the suitability of genetic barcodes to trace CRISPR-derived GMOs without affecting the performance of the resulting strains.

**Supplementary Information:**

The online version contains supplementary material available at 10.1007/s00294-021-01164-5.

## Introduction

Over the last decades, genetically modified organisms (GMOs) have revolutionized many fields, including medicine, agriculture, food or pharmaceutical industries (Hug 2008). Genetic engineering is a powerful tool for the production of metabolites or enzymes, controlling/directing the synthesis of desired products, or for basic biological research. Despite the fact that gene transfer occurs naturally (Gogarten and Townsend 2005), there is a social concern about the impact of GMOs in animal/human health and the environment due to the unpredicted pleiotropic effect of foreign genes or the possible loss of genetic diversity (Font 2011). This explains why the application of genetic engineering for strain improvement is strictly controlled with an extensive legal framework, risk management and assessment procedures (Pavone et al. 2011).

Safe manipulation of genome-edited strains implies the ability to track the presence of these strains in any of the environments they are utilized, especially if using pathogens or other harmful strains that could escape into the environment. In this context, introducing genetic barcodes within the editing site represents a valuable tool as "fingerprints" for the identification of microbial strains that have undergone genetic modifications. DNA barcodes have been long used as a method for the identification of microorganisms using a short section of specific DNA present in the genome, e.g., the 16S rRNA gene in the case of prokaryotes (Valentini et al. 2009), or the 18S rRNA gene in the case of eukaryotes (Piganeau et al. 2011). Additionally, genetic barcodes have been applied for the identification of pathogens of quarantine importance to Europe, which are collected in the Q-bank database (www.q-bank.eu). However, these barcodes are specific genetic regions already present in the genomes, and the effects of introducing exogenous barcodes for the identification of genetically modified strains have been barely addressed so far.

The bacterial and archaeal immune mechanism known as Clustered Regularly Interspaced Short Palindromic Repeats (CRISPR)/CRISPR associated protein 9 (Cas9) has been engineered into a powerful genome-editing tool (Jinek et al. 2012). Due to its efficiency, versatility and simplicity, CRISPR/Cas9 has become one of the fastest-growing genome-editing technologies and revolutionized modern science and genetic studies in a wide range of organisms, including human cell lines (Cong et al. 2013; Mali et al. 2013), fish (Hwang et al. 2013), plants (Belhaj et al. 2013), yeasts (DiCarlo et al. 2013), and filamentous fungi (Deng et al. 2017; Kun et al. 2019). CRISPR/Cas9 allows for precise edits to DNA in living cells, with the possibility to generate clean and scarless modifications. This makes CRISPR-derived GMOs very difficult (or almost impossible) to distinguish from strains with naturally occurring mutations or other genetically modified (non-GMO) strains obtained by random mutagenesis. Nevertheless, CRISPR-derived (micro) organisms fall under the strict GMO regulations in the European Union (Directive 2001/18/EC), which implies cost- and labor-intensive pre-market evaluations and long approval processes before commercialization.

Filamentous fungi are a large group of eukaryotic organisms that can grow in many different environments and have the ability to produce a wide range of enzymes and metabolites, arousing high interest for the scientific community for their exploitation as cell factories in biotechnology (Meyer 2008). However, fungi can also deteriorate buildings, spoil food, or produce undesirable metabolites such as mycotoxins with hazardous effects on plants, animals and human health (Tola and Kebede 2016). *Aspergillus* sp., especially *Aspergillus niger,* are among the most widely used fungi for industrial applications (Baker 2006; Frisvad et al. 2018), and several of its metabolites and enzymes are "Generally Recognized As Safe" (GRAS) by the American Food and Drug Administration (FDA) (Schuster et al. 2002). Efficient CRISPR/Cas9 methods have been developed for the genetic modification of Aspergilli (Nødvig et al. 2015), and especially for *A. niger* (Kun et al. 2020; Song et al. 2018), improving the utility of *A*. *niger* as an industrial cell factory and as a reference organism for research.

In this study, we introduced a short (20 bp) and unique genetic barcode within the editing site of genetically modified strains obtained by CRISPR/Cas9 that allows their identification as CRISPR-derived GMOs and the traceability of these mutants. For this, we used *A. niger* as a test case and deleted two genes encoding two well-studied transcription factors (TFs) involved in plant biomass degradation, the xylanolytic and arabinanolytic TFs XlnR and AraR, respectively (Battaglia et al. 2011; van Peij et al. 1998). We studied and compared the efficiency of the genetic modification and the traceability of barcoded and non-barcoded strains using PCR, which is a routinely used screening technique in laboratories of molecular biology, and provides advantages to the use of more sophisticated, time-consuming and expensive techniques such as genome sequencing or real-time PCR (RT-qPCR) for strain identification. Additionally, possible differences in growth and protein production due to the presence of barcodes were assessed.

## Material and methods

### Strains, media and culture conditions

*Escherichia coli* DH5α was used for vector propagation, and was grown in Luria–Bertani (LB) medium (Bertani 1952) supplemented with 50 μg/mL ampicillin (Sigma-Aldrich).

*Aspergillus niger* CBS 138852 is a progeny of CBS120.49 and was used as parent strain for subsequent transformation events. The generated *A. niger* mutants were deposited at the culture collection of Westerdijk Fungal Biodiversity Institute and are shown in Table 1.

**Table 1:** *A. niger* strains used in this study

Strain	CBS number	Genotype	Barcode	Reference
Reference	CBS 138852	N593, *cspA1*, *kusA*::*amdS*, *pyrG* ^−^	No	(Meyer et al. 2007)
Δ*xlnR*	CBS 145447	N593, *cspA1*, *kusA*::*amdS*, *pyrG* ^−^, Δ*xlnR*	Yes	(Kun et al., unpublished)
Δ*araR*	CBS 145451	N593, *cspA1*, *kusA*::*amdS*, *pyrG* ^−^, Δ*araR*	Yes	(Kun et al., unpublished)
Δ*xlnR*	CBS 147370	N593, *cspA1*, *kusA*::*amdS*, *pyrG* ^−^, Δ*xlnR*	No	This study
Δ*araR*	CBS 147369	N593, *cspA1*, *kusA*::*amdS*, *pyrG* ^−^, Δ*araR*	No	This study

For strain propagation, *A. niger* strains were routinely cultured in *Aspergillus* Minimal-Medium (MM) or Complete Medium (CM) at 30℃ (de Vries et al. 2004) supplemented with 1% d-glucose and 1.22 g/L uridine (Sigma-Aldrich). Conidia were harvested, dispersed in *N*-(2-acetamido)-2-aminoethanesulfonic acid (ACES) buffer (Good 1966), and concentration was adjusted using a haemocytometer.

Growth profiles were carried out using MM containing 25 mM d-glucose, d-xylose, l-arabinose (Sigma-Aldrich), or 1% beechwood xylan or wheat bran. All media were supplemented with 1.22 g/L uridine. Plates were inoculated in duplicate with a 2-µL suspension containing 10^3^ conidia and incubated at 30˚C for up to 6 days. Growth was monitored daily by visual inspection.

### DNA construction, fungal transformation and mutant purification

The ANEp8-Cas9-*pyrG* plasmid containing the autonomous fungal replicating element AMA1 (Gems et al. 1991), *pyrG* as selection marker, *cas9* gene, and the single guide RNA (gRNA) expression construct under the control of the proline transfer ribonucleic acid (tRNA^Pro1^) promoter was used in this study (Kun et al. 2020; Song et al. 2018) and propagated in *E. coli*. The design of the protospacers (20 bp) for the gRNAs was performed using the Geneious 11.04.4 software (https://www.geneious.com). The gRNA sequences (Table 2) were designed based on the experimentally determined predictive model described by Doench et al. 2014. The gRNAs for each gene were obtained to delete *araR* (Gene ID: NRRL3_07564) and *xlnR* (Gene ID: NRRL3_04034) genes in *A. niger* CBS 138852 after repair of the double-strand breaks (DSBs) caused by Cas9. All repair templates (RTs) include the 5′ and 3′ flanking regions of the target genes for homologous recombination, and in some cases included the 5′-ACTGCTAGGATTCGCTATCG-3′ genetic barcode for traceability (Table 2). The barcode was designed based on the following criteria: (1) it is a random, non-coding sequence composed of 20 nucleotides, a length that matches the standards of common PCR primers; and (2) the nucleotide sequence was chosen due to its lack of homology with the genomic sequence of *A. niger* NRRL3, which is the ancestor of *A. niger* N593 strain used in this study (Table 1). The RTs were obtained by fusion-PCR using Phusion™ High-Fidelity DNA Polymerase (Thermo Fisher Scientific) following the manufacturer’s instructions. Two PCR fragments were generated by amplifying approx. 750 bp upstream and downstream of the *araR* and *xlnR* genes. These two fragments were fused together in a second fusion-PCR obtaining approx. 1200 bp RT, and were subsequently purified using the Wizard® SV Gel and PCR Clean-Up System (Promega).

**Table 2:** Primers used in this study

Primer Number	Primer ID	Use	TM (ºC)	Sequence (5′ → 3′)^a, b^	Purpose^c^
–	P1-gRNA	F	57.3	CAACCTCCAATCCAATTTGACTCCGCCGAACGTACTG	gRNA
–	P2-gRNA	R	54.1	ACTACTCTACCACTATTTGAAAAGCAAAAAAGGAAGGTACAAAAAAGC	gRNA
–	P3-*xlnR*	R	53.2	**CGGTCTCCTGGCGAGTATGC**GACGAGCTTACTCGTTTCG	gRNA
–	P4-*xlnR*	F	49.3	**GCATACTCGCCAGGAGACCG**GTTTTAGAGCTAGAAATAGCAAG	gRNA
–	P3-*araR*	R	53.2	**CCCAGAAAGTCAGGGCACAC**GACGAGCTTACTCGTTTCG	gRNA
–	P4-*araR*	F	49.3	**GTGTGCCCTGACTTTCTGGG**GTTTTAGAGCTAGAAATAGCAAG	gRNA
–	Fw-screen	F	47.9	TTTTCTCTTCCATTTACGC	cse
–	Rev-screen	R	53.1	GGGGATCATAATAGTACTAGCCA	cse
199	An-*xlnR*_5F	F	55.4	GTGTGTGTGTGAGAGAGAAAGG	RT, csa
200	An-*xlnR*_5R	R	55.1	GCATCTCATCATCAGCCGTGTGGAAAGTGAGGTATTCAGACCG	RT
201	An-*xlnR*_3F	F	57.6	CGGTCTGAATACCTCACTTTCCACACGGCTGATGATGAGATGC	RT
202	An-*xlnR*_3R	R	57.2	GACGAGAGGAGTTGGTAGCG	RT, csa
4	An-*xlnR*_5R_BC	R	55.1	*CGATAGCGAATCCTAGCAGT*GGAAAGTGAGGTATTCAGACCG	RT (BC)
5	An-*xlnR*_3F_BC	F	57.6	*ACTGCTAGGATTCGCTATCG*ACACGGCTGATGATGAGATGC	RT (BC)
15	An-*xlnR* NEST_5F	F	55.7	CTTTCTCGTGGGTTCTTCACC	RT
204	An-*xlnR* NEST_3R	R	56.6	GGATGTAGTCGTCCAGGAGG	RT
193	An-*araR* _5F	F	59.2	GTCCGCAAGTTGTGTGGTGG	RT, csa, TR
194	An-*araR* _5R	R	56.2	GCATCGGTGCTGTGAGAAACGGAATCGCAGTCTGATGAAACG	RT
195	An-*araR* _3F	F	56.7	CGTTTCATCAGACTGCGATTCCGTTTCTCACAGCACCGATGC	RT
196	An-*araR* _3R	R	57.7	AACCGAGAAGCCCAGTTTCG	RT, csa, TR
41	An-*araR* _5R_BC	R	56.2	*CGATAGCGAATCCTAGCAGT*GGAATCGCAGTCTGATGAAACG	RT (BC)
42	An-*araR* _3F_BC	F	56.7	*ACTGCTAGGATTCGCTATCG*GTTTCTCACAGCACCGATGC	RT (BC)
197	An-*araR* NEST_5F	F	57.8	GAAGCGACCTCATAGCGACC	RT
198	An-*araR* NEST_3R	R	56.7	ATGCCAGAAACATGCGATGC	RT
1	Dlinker-F	F	54.0	ACTGCTAGGATTCGCTATCG	csa
2	Dlinker-R	R	54.0	CGATAGCGAATCCTAGCAGT	csa, TR
48	*araR*_intern-F	F	56.8	CAAACCGTTTCATCAGACTGCG	TR
207	5′F *araR*Fus	F	53.8	CTGCGATTCCGTTTCTCAC	csa, TR
228	3′R *araR*Fus	R	50.9	GCTGTGAGAAACGGAATC	csa, TR
209	5′F *xlnR*Fus	F	56.2	CTCACTTTCCACACGGCTG	csa
210	3′R *xlnR*Fus	R	56.2	CAGCCGTGTGGAAAGTGAG	csa

*F* forward, *R* reverse, *TM* temperature of melting

^a^The gRNA sequence is highlighted in bold

^b^Barcode is represented in italics

^c^gRNA single guide, RNA RT: repair template, BC with barcodes, TR traceability, cse: colony screening for *E. coli*, csa colony screening for *A. niger*

CRISPR/Cas9 plasmid construction, generation of *A. niger* protoplasts, genetic transformation and purification of transformants were performed as previously described (Kun et al. 2020). Genomic DNA was obtained from fungal mycelia using Wizard® Genomic DNA Purification kit (Promega). Mutant strains were confirmed by PCR through the amplification of target gene region (Figs. S1, S2). All primers used in this study are shown in Table 2 and were ordered from Integrated DNA Technologies (IDT, Leuven, Belgium).

### Protein production assays

For protein production analysis and comparison, 10^6^ conidia/mL of *A. niger* parental strain, two independent barcoded ∆*araR*, and two non-barcoded ∆*araR* mutant strains were pre-cultured in 250 mL CM containing 2% d-fructose and 1.22 g/L uridine for 16 h at 30˚C and 250 rpm. Mycelial aliquots (~ 2.5 g wet weight) were then transferred to 250-mL Erlenmeyer flasks containing 50 mL MM supplemented with 1.22 g/L uridine and with 1% l-arabinose or 1% wheat bran as inducing conditions for protein production, and 1% d-fructose as non-inducing condition. Flasks were incubated at 30˚C and 250 rpm. Supernatant samples were taken after 24 and 48 h of incubation and centrifuged for 10 min at 13,500 × *g*. Twelve μL of each sample were analyzed by SDS-PAGE using SDS-12% polyacrylamide gels calibrated with PageRuler™ Plus Prestained Protein Ladder (Thermo Scientific) and silver stained. Samples were evaluated in biological duplicates.

### Traceability assays

For the traceability of the *A. niger* parental, barcoded and non-barcoded Δ*araR* strains on DNA mixtures containing both parental and mutant genomic DNA at specific concentrations, strains were statically grown at 30ºC in liquid MM with 1% d-glucose and supplemented with 1.22 g/L uridine prior to DNA isolation. Genomic DNA was isolated from 5-day-old mycelia using Wizard® Genomic DNA Purification kit (Promega) and the DNA concentration was measured with NanoDrop ND-1000 (Thermo Scientific). Controlled amounts of genomic DNA from the parental and mutant strains were mixed in different ratios and amplification reactions (Table 3), and PCRs were performed using GoTaq® Flexi DNA polymerase (Promega) according to the manufacturer's instructions using primers shown in Table 2.

**Table 3:** Genomic DNA mixtures used for the traceability of the parental, barcoded and non-barcoded ∆*araR* strains

Condition	Parental DNA (ng)	Mutant DNA (ng)
1	10	10
2	10	1
3	10	0.1
4	10	0.01
5	10	0.001
6	10	0

For the traceability of the *A. niger* parental, barcoded and non-barcoded Δ*araR* strains in mycelia originated from mixed conidia populations, controlled conidia concentrations of the parental and mutant strains were mixed (Table 4) in 250-mL Erlenmeyer flasks with 50 mL of CM with 1% d-glucose and 1.22 g/L uridine. Flasks were incubated at 30ºC and 200 rpm. After 24 h of growth, mycelia were harvested by vacuum filtration through sterile cheesecloth, rinsed with sterile Milli-Q water, and frozen in liquid nitrogen. Genomic DNA was isolated using Wizard® Genomic DNA Purification kit (Promega). DNA concentration was measured with NanoDrop ND-1000 (Thermo Scientific) and several quantities of DNA (50, 10, 1 and 0.1 ng) were used in different amplification reactions performed with GoTaq® Flexi DNA polymerase (Promega) according to the manufacturer's instructions using primers shown in Table 2. As control, 0.1 ng genomic DNA obtained from mycelia coming from different conidia concentrations of the mutant strains alone (with no parental DNA) were used (Table 4). Two biological replicates were used per condition tested.

**Table 4:** Conidia mixtures used for the traceability of the parental, barcoded and non-barcoded ∆*araR* strains

Condition	Parental (conidia/mL)	Mutant (conidia/mL)
1	10^6^	10^6^
2	10^6^	2 × 10^5^
3	10^6^	10^5^
4	10^6^	2 × 10^4^
5	10^6^	10^4^
6	10^6^	10^3^
7	10^6^	10^2^
8	10^6^	0
C1	0	10^6^
C2	0	2 × 10^5^
C3	0	10^5^
C4	0	2 × 10^4^
C5	0	10^4^
C6	0	10^3^
C7	0	10^2^

*C* control

## Results and discussion

### The presence of barcodes does not influence transformation efficiency or growth of* A. niger*

**Fig. 1:**
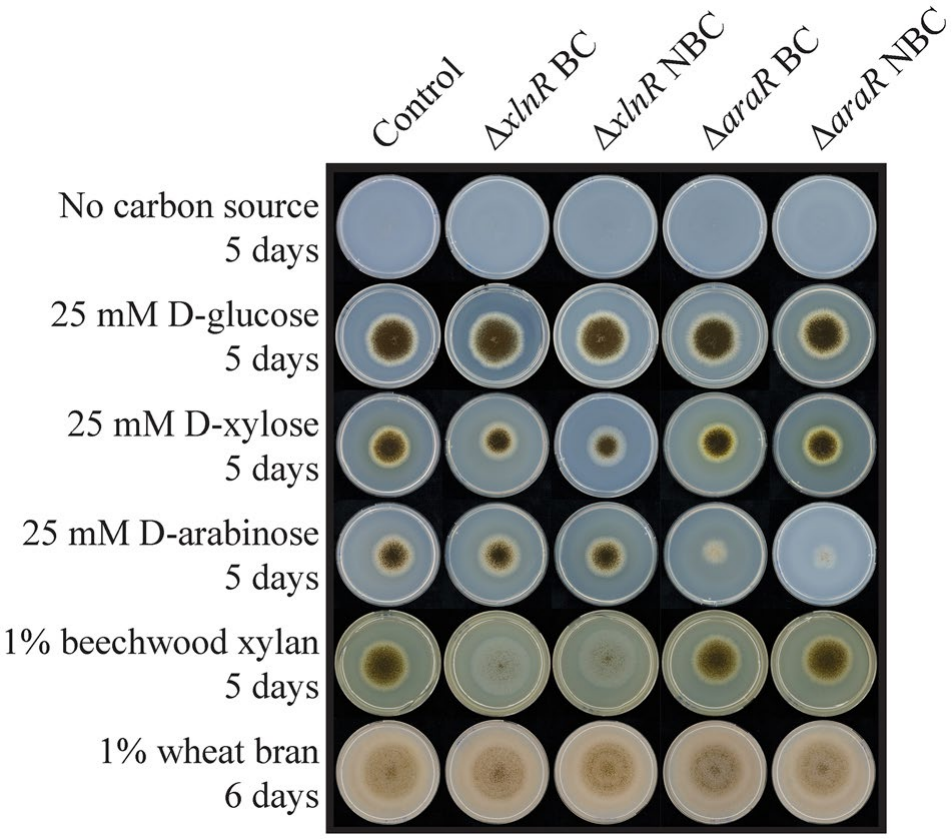
Growth profiles of the parental, barcoded and non-barcoded ∆*araR* and ∆*xlnR A*. *niger* strains. Strains were grown up to 6 days at 30ºC. BC: with barcodes; NBC: without barcodes

In this study, barcoding is defined as the insertion of a 20-bp nucleotide sequence within the genome-editing sites that can be used to identify and track genetically modified strains. However, it is barely known whether the introduction of these sort of barcodes might influence the efficiency of the genetic transformation or would result in unexpected effects on the resulting strains. For this reason, the possible effects of the insertion of exogenous barcodes in CRISPR/Cas9-edited strains have been evaluated in *A. niger* CBS 138852. We transformed *A. niger* to generate both barcoded and non-barcoded Δ*araR* and Δ*xlnR* strains using CRISPR/Cas9 in one go as previously described (Kun et al. 2020). In all cases, the gRNAs used for the generation of barcoded and non-barcoded strains with a given genetic modification (Δ*araR* or Δ*xlnR*) was the same, and only RTs were different (Figs. S1, S2). After protoplast transformation, the number of colonies obtained on the transformation plates that contained the barcode (11) was comparable to that of the transformant strains without barcode (20), and such small differences in these numbers are attributed to experimental variation. Strains were finally confirmed by PCR (Figs. S1, S2). With these results, we conclude that the introduction of barcodes does not influence the efficiency of the transformation in *A. niger* by using the CRISPR/Cas9 technology*.* To further investigate the possible unexpected effects on the fitness of the resulting strains, we also studied the influence of the barcodes on growth on selected substrates (Fig. 1).

All parental and mutant strains grew equally well on d-glucose, which is expected as deletion of AraR or XlnR does not affect growth on this sugar. Similarly, all strains grew equally well on wheat bran, which is a complex substrate rich in arabinoxylan and cellulose. As expected, Δ*araR* strains grew poorly on l-arabinose, while Δ*xlnR* strains showed a slightly reduced growth on d-xylose and abolished growth on xylan. These results can be explained by the reduced ability of Δ*araR* and Δ*xlnR* to utilize L-arabinose and d-xylose, respectively, since AraR and XlnR co-regulate the metabolic genes involved in pentose catabolism in Aspergilli (Battaglia et al. 2014). Also, abolished growth on xylan is explained by the inability of Δ*xlnR* strains to produce xylanases, which are responsible for breaking the xylan polymeric backbone (de Vries and Visser 2001). However, in all cases the phenotypic behavior of barcoded and non-barcoded strains are the same, demonstrating that the presence of barcodes at the editing sites does not influence growth of the resulting strains.

### The presence of barcodes does not influence protein production in* A. niger*

**Fig. 2:**
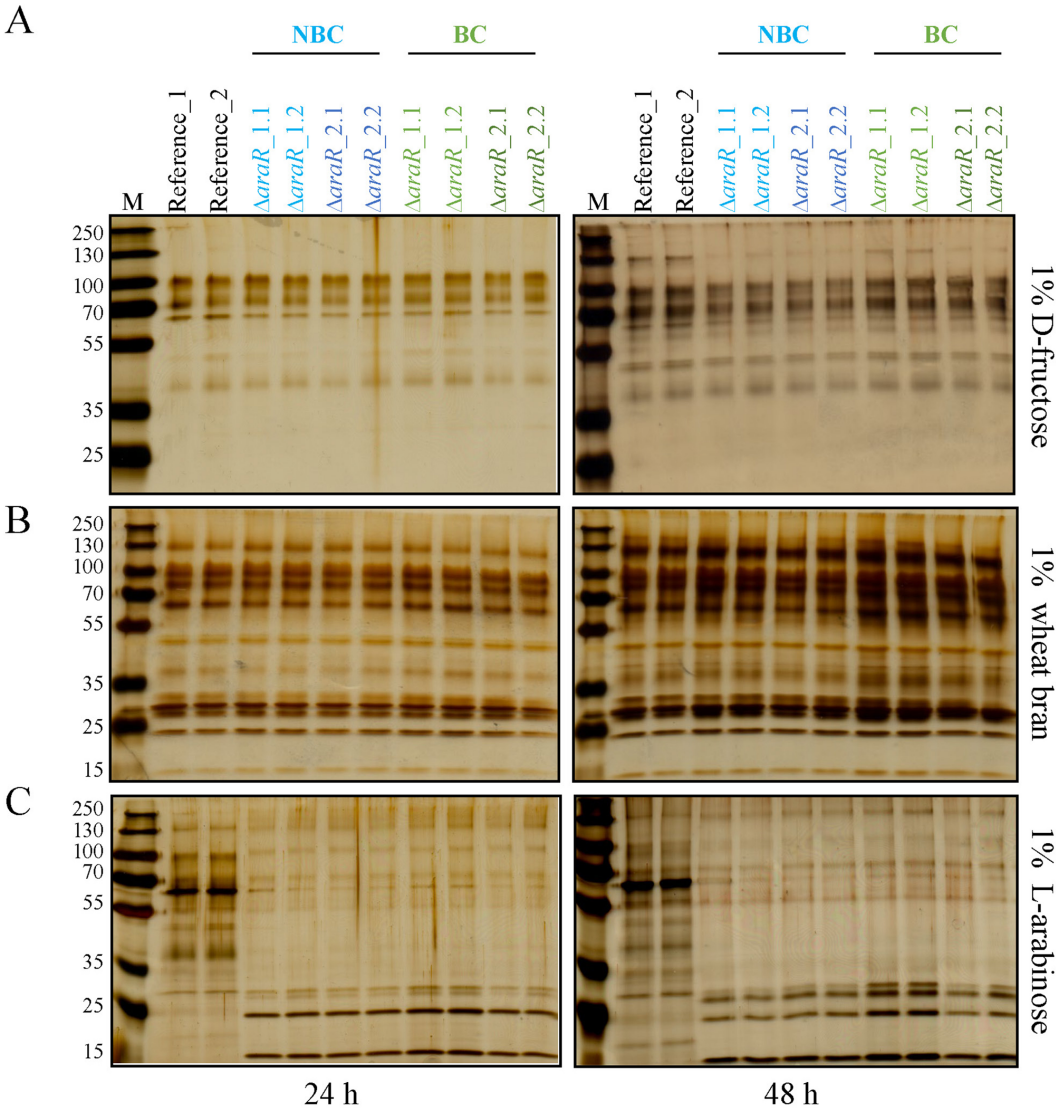
Protein production analysis of barcoded and non-barcoded ∆*araR* strains obtained by CRISPR/Cas9. SDS-PAGE analysis of supernatants of two independent non-barcoded (NBC, blue) and barcoded strains (BC, green) after 24 and 48 h of growth in MM + 1% d-fructose (**a**), 1% wheat bran (**b**) and 1% l-arabinose (**c**). Twelve μL of each supernatant were loaded per well. Two biological replicates are shown per strain. M: PageRuler™ Plus Prestained Protein Ladder (Thermo Scientific)

In the previous section, we showed that the presence of exogenous genetic barcodes within the editing site in *A. niger* Δ*araR* and Δ*xlnR* neither influenced transformation efficiency nor growth ability of these mutants on several carbon sources. As similar results were obtained for *araR* and *xlnR* and the barcode nucleotide sequence introduced in both barcoded Δ*araR* and Δ*xlnR* was the same (Table 2), we chose barcoded and non-barcoded Δ*araR* as reference for further characterization regarding protein production and traceability of the genetically modified strains. To further investigate whether the introduction of barcodes affects the performance of the resulting strains, protein production in the barcoded Δ*araR* was studied and compared to that of the non-barcoded Δ*araR* in liquid cultivation. For this, the extracellular protein production of two independent barcoded Δ*araR* and two non-barcoded Δ*araR* mutants were analyzed by SDS-PAGE after 24 and 48 h of growth under different conditions (Fig. 2).

Transcription factors play a key role controlling the production of the specific enzymes required to degrade the different plant polymers. Some TFs also participate in the catabolism of the sugars released after plant biomass degradation, and are thus induced under the presence of these sugars (Benocci et al. 2017). For this reason, to compare *A. niger* barcoded and non-barcoded Δ*araR* strains ability to produce enzymes, three different carbon sources were used for different levels of induction. d-fructose was used as control of non-inducing condition for polysaccharide-degrading enzymes production. In contrast, wheat bran and l-arabinose were used to induce the production of polysaccharide-degrading enzymes in *A. niger*.

As expected, all strains showed low protein production levels when they were grown in 1% d-fructose (Fig. 2, top panels). Protein production was much more abundant under inducing conditions for all the strains grown in 1% wheat bran (Fig. 2, middle panels). Protein patterns between Δ*araR* mutants and the reference strain differ significantly when growing on l-arabinose as sole carbon source (Fig. 2, bottom panels) due to the inability of Δ*araR* mutants to utilize l-arabinose as carbon source and induce enzyme production in response to this sugar (Battaglia et al. 2014; de Groot et al. 2007).

Protein production was higher after 48 h of growth in all the strains and conditions tested, and more importantly, protein patterns were very similar among barcoded and non-barcoded strains in the three different carbon sources tested after 24 and 48 h. These results demonstrate that the presence of the 20-bp exogenous barcode within the editing site, in this case within the *araR* locus, does not (negatively) influence protein production in *A. niger*, and thus, these strains are comparable.

### Barcoded and non-barcoded mutants can be similarly detected in DNA mixtures

**Fig. 3:**
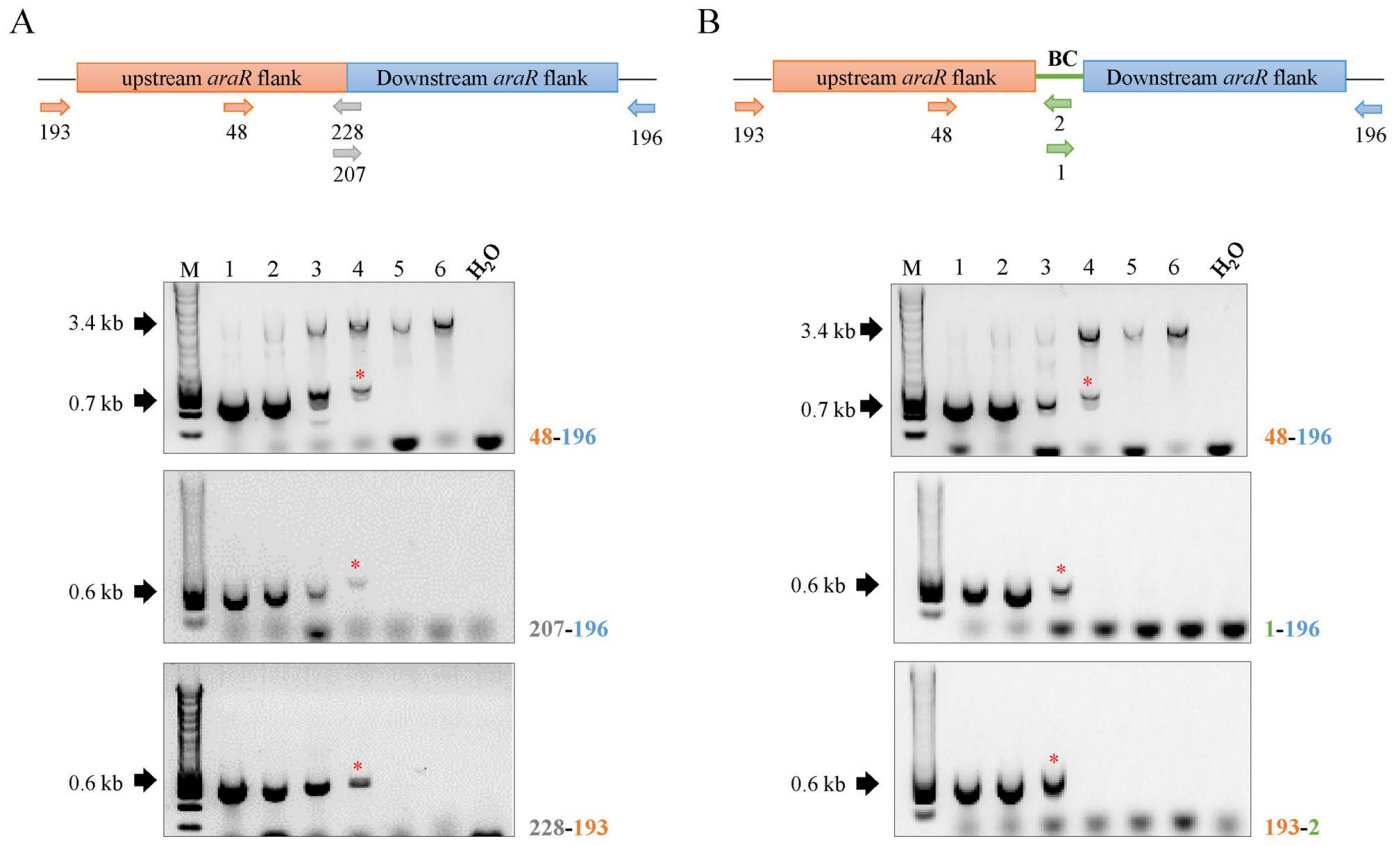
Study of the traceability of the mutants in genomic DNA populations.** a** Non-barcoded mutants. **b** Barcoded mutants. Numbers in each well correspond to the DNA mixtures shown in Table 3. Primer locations are shown on the upper schemes. Primer pair combination for each PCR reaction is shown next to the corresponding electrophoresis gel. Lower bands (0.6, 0.7 kb) correspond to the mutants, whereas the higher bands (3.4 kb) correspond to the reference strain. Asterisk (*) represents the lowest tested concentration in which the mutant can be detected. M: molecular weight marker (HyperLadder™ 1 kb, Bioline). Figures are not drawn to scale

Decreasing amounts of genomic DNA from the barcoded and non-barcoded ∆*araR* mutants were mixed with a fixed amount of gDNA from the parental strain (Table 3) in order to determine up to which condition mutants can be detected by PCR in DNA pools where the ratio of parental/mutant genomic DNA is known. In case of the non-barcoded ∆*araR* mutants, the *araR* 5′ upstream and 3′ downstream flanking regions were fused together (Fig. S1a), and two specific primers were designed on the fusion site (207, 228) (Fig. 3a). Three different primer pairs (48-196; 207-196; 228-193) (Table 2) were used in three independent PCR reactions. In the case of the barcoded mutants (Fig. 3b), the *araR* 5′ upstream and 3′ downstream flanking regions were fused to the barcode (5′-ACTGCTAGGATTCGCTATCG-3′) (Fig. S1b), and two specific primers where designed in the barcode site (1, 2, in green) (Fig. 3b). As for the non-barcoded mutants, three different primer pairs (48-196, 1-196, 193-2) were used in three independent PCRs. In both cases, the specific location and orientation of the primers are shown in Fig. 3 (top panels). Primers 48 and 196 are located at the 5′ and 3′ flanking regions of the *araR* gene, respectively, allowing the detection of both the parental (3.4 kb band) and the mutant (0.7 kb band) strains in the same amplification reaction regardless of the presence of a barcode. In contrast, the other primer pairs used only allow the identification of either the barcoded or non-barcoded ∆*araR* mutants (0.6 kb bands), where the *araR* gene is absent.

Results indicate that barcoded and non-barcoded mutants are similarly traceable, and DNA pools containing between 0.1 and 0.01 ng of mutant DNA still allow the traceability of the barcoded and non-barcoded mutants, which corresponds to a ratio of parental/mutant DNA of 1:100–1:1000. However, DNA amounts of 0.001 ng or lower do not allow the detection of the mutants under our working conditions. In addition, primers designed in the fusion site of the non-barcoded mutants (207-228) (Fig. 3a) are more efficient than the primers designed in the barcoding-site (1-2) **(**Fig. 3b), confirming the importance of primer design, primer efficiency and optimization of the PCR conditions for the successful detection of the DNA region of interest (Mallona et al. 2011). Moreover, these results are independent of the presence of parental DNA in the mixtures, since these results are also reproduced when no parental DNA is added to the PCRs (Fig. S3).

### Barcoded and non-barcoded mutants can be similarly detected in mycelia originated from mixed conidia populations

Safe use of CRISPR/Cas9 genome-edited strains requires the ability to track the presence of the generated strains in any of the environments they are utilized. Our previous results showed that barcoded and non-barcoded mutants are similarly traceable in controlled parental/mutant genomic DNA mixtures, and that DNA amounts between 0.01 and 0.001 ng still allowed the traceability of both barcoded and non-barcoded mutants by PCR.

**Fig. 4:**
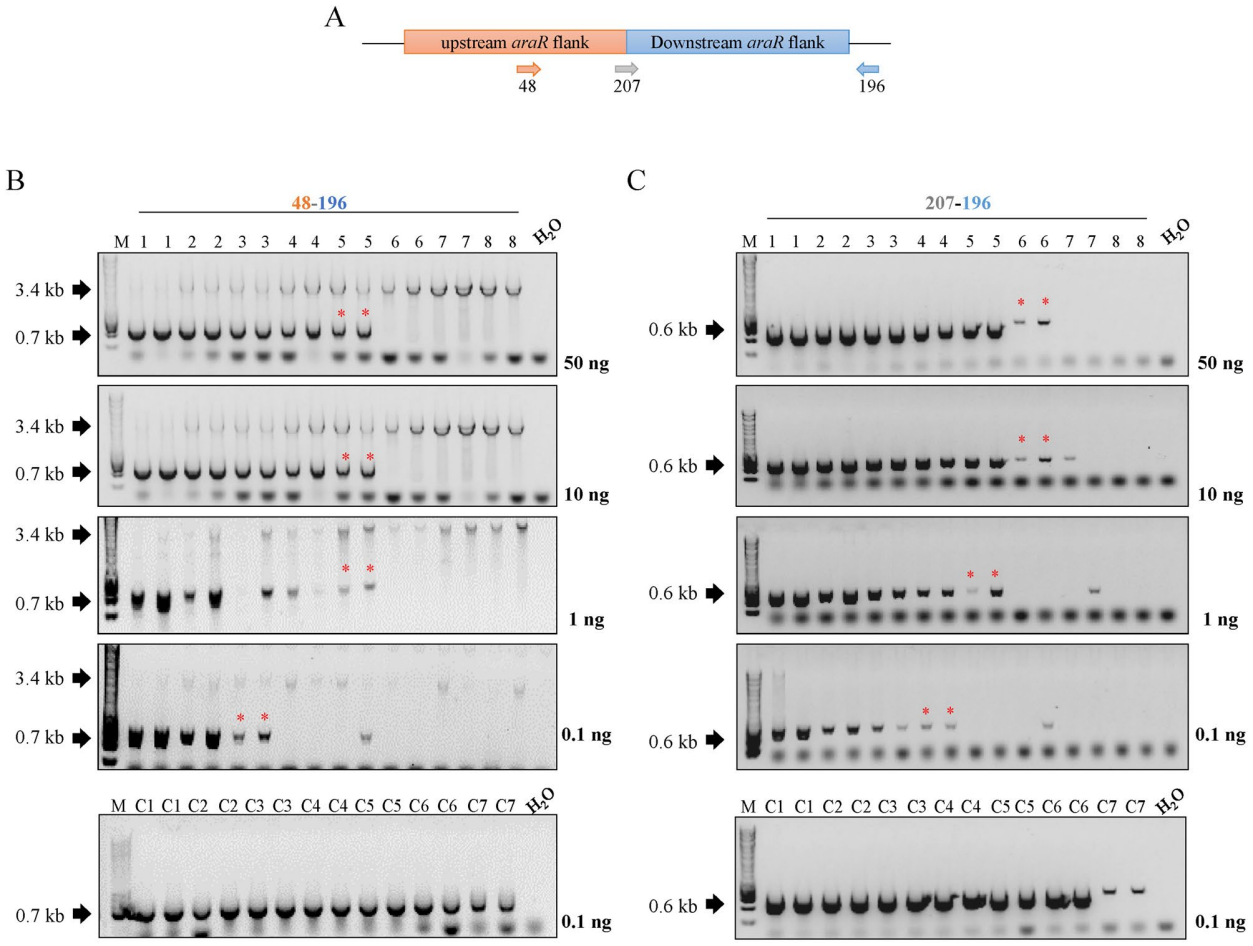
Study of the traceability of non-barcoded mutants in conidia populations.** a** Schematic representation of the primer locations in the non-barcoded ∆*araR* mutants. **b** PCR results for the traceability of the non-barcoded ∆*araR* mutants using primer pair 48-196. **c** PCR results for the traceability of the non-barcoded ∆*araR* mutants using primer pair 207-196. Lower bands (0.6, 0.7 kb) correspond to the mutants, whereas the higher bands (3.4 kb) correspond to the reference strain. The numbers in the wells correspond to the conidia combinations shown in Table 4. Amount of DNA used for each analysis is shown next to the corresponding electrophoresis gel. Asterisk (*) represents the lowest tested concentration in which the mutant can be detected. M: molecular weight marker (HyperLadder™ 1 kb, Bioline)

**Fig. 5:**
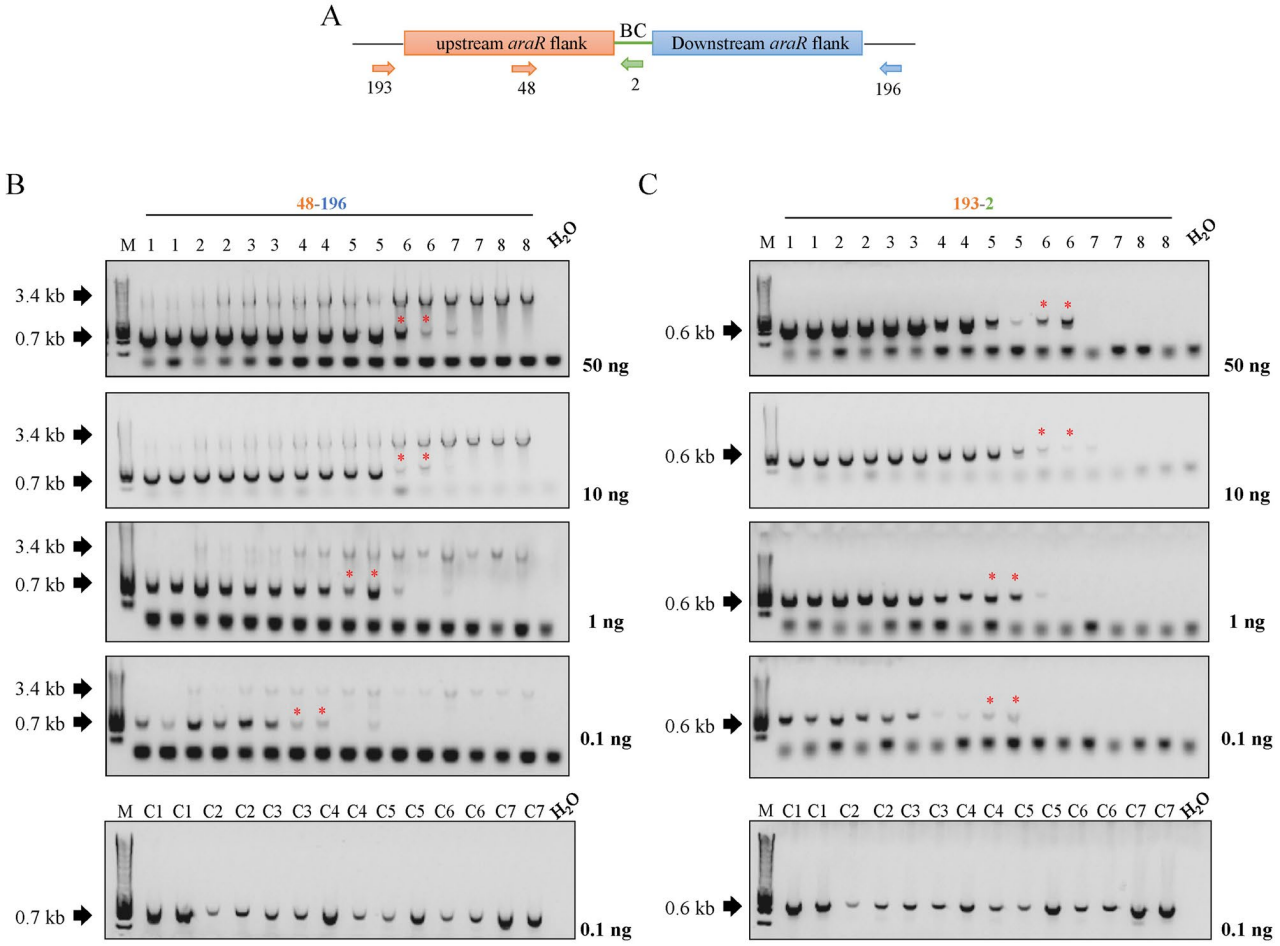
Study of the traceability of barcoded mutants in conidia populations.** a** Schematic representation of the primer locations in the barcoded ∆*araR* mutants. **b** PCR results for the traceability of the barcoded ∆*araR* mutants using primer pair 48-196. **c** PCR results for the traceability of the barcoded ∆*araR* mutants using primer pair 207-196. Lower bands (0.6, 0.7 kb) correspond to the mutants, whereas the higher bands (3.4 kb) correspond to the reference strain. The numbers in the wells correspond to the conidia combinations shown in Table 4. Amount of DNA used for each analysis is shown next to the corresponding electrophoresis gel. Asterisk (*) represents the lowest tested concentration in which the mutant can be detected. M: molecular weight marker (HyperLadder™ 1 kb, Bioline)

In nature, fungi reproduce by spreading microscopic spores, and these spores are often present in the air and soil, where they enter into contact with many other different species. In order to mimic a more natural scenario, we also wanted to study the traceability of barcoded and non-barcoded strain in different parental/mutant mycelial mixture originated from conidia populations at a laboratory scale. For this aim, decreasing amounts of conidia from the barcoded and non-barcoded ∆*araR* mutants were mixed with a fixed amount of parental conidia (Table 4) in 50 mL of growth medium. Mycelia were harvested after 24 h of growth and genomic DNA was subsequently isolated. In contrast to DNA populations, where the proportion of genomic DNA of both parental and mutant strains is known (see Sect. Barcoded and non-barcoded mutants can be similarly detected in DNA mixtures), in this case we can only measure the total amount of DNA present in the sample isolated from the mycelia mixtures. To determine at which dilution the genome-edited strains can be identified and to compare the efficiency of detecting the actual mutation versus detecting the barcode, several quantities of DNA (50, 10, 1, and 0.1 ng) were used in different amplification reactions using different primer pairs (Figs. 4, 5; Table 2). Since previous results in DNA mixtures showed that between 0.1 and 0.01 ng DNA still allowed the detection of the mutants, we chose 0.1 ng as the minimum amount of DNA we used in the subsequent PCRs to ensure successful detection.

In case of the non-barcoded mutants (Fig. 4), two primer pairs (48-196 and 207-196) were used in independent PCRs. When using primer pair 48-196, which allows the detection of both parental and mutant strains in the same PCR, the mutant DNA can be detected up to condition 5 when 50, 10 and 1 ng DNA are applied in the PCRs (Fig. 4b), which corresponds to a parental/mutant conidia concentration of 10^6^/10^4^ conidia/mL (Table 4). However, the sensitivity of mutant detection drops until parental/mutant conidia concentration of 10^6^/10^5^ conidia/mL when 0.1 ng of DNA are used (condition 3, Table 4). On the other hand, primer pair 207-196 is specific for the detection of the non-barcoded mutant strain. With these primers, the non-barcoded mutant was detected up to condition 6 when either 50 or 10 ng DNA were used (Fig. 4c). This corresponds to parental/mutant conidia concentration of 10^6^/10^3^ conidia/mL (Table 4), and sensitivity of mutant detection drops gradually with decreasing DNA concentration until parental/mutant conidia concentration of 10^6^/ 2 × 10^4^ conidia/mL when 0.1 ng DNA were used (condition 4, Table 4). As control, 0.1 ng DNA obtained from mycelia from different conidia concentrations of the mutant strains alone were used for PCR with the two primer pair combinations used. In all cases, the mutant strain could be detected up to condition C7 (Fig. 4, bottom panels; Table 4).

Similarly, primer pairs 48-196 and 193-2 were used to track the barcoded ∆*araR* mutant. With primer pair 48-196, mutant DNA can be detected up to condition 6 when using either 10 or 50 ng of DNA in the PCRs, which corresponds to a parental/mutant concentration of 10^6^/10^3^ conidia/mL (Fig. 5b; Table 4). However, sensitivity of mutant detection drops gradually with the decrease of DNA concentration until a parental/mutant conidia concentration of 10^6^/ 2 × 10^4^ conidia/mL when 0.1 ng DNA were used. These results are consistent between barcoded and non-barcoded strains, since this primer pair allows the detection of both parental and mutant strains regardless the presence of barcodes. On the other hand, primer pair 193-2 is specific for the detection of the barcoded mutants, since primer 2 is located specifically at the barcode site (Fig. 5a). With these primers, the barcoded strain was detected up to condition 6 when either 50 or 10 ng DNA were used (Fig. 5c). This corresponds to parental/mutant conidia concentration of 10^6^/10^3^ conidia/mL (Table 4), and sensitivity of mutant detection drops gradually with the decrease of DNA concentration until parental/mutant conidia concentration of 10^6^/ 10^4^ conidia/mL when 0.1 ng DNA were used. As control, 0.1 ng genomic DNA obtained from mycelia from different conidia concentrations of the mutant strain alone were used for PCR amplification with the two primer pair combinations used. In all cases, the mutant strain could be detected up to condition C7 (Fig. 5, bottom panels; Table 4).

Overall, these results demonstrate that both barcoded and non-barcoded mutants can be easily tracked in conidia populations under the conditions tested. Additionally, barcoded and non-barcoded strains can be similarly detected after 24 h of growth in a ratio up to 1:1000 (parental/mutant conidia) when either 50 or 10 ng DNA was used in the PCRs, regardless of the primers used. Nevertheless, these tests can only be applied when the specific CRISPR/Cas9 modification is known. Tracing non-disclosed CRISPR/Cas9 events will be nearly impossible unless (i) the parent strain is also available, in which case re-sequencing could indicate possible CRISPR/Cas9 modifications, or (ii) the identity of the barcode sequences is known and universally applied. In this case, simple PCRs using primers located at the barcode sites will allow the identification of a CRISPR/Cas9 mutant without the need to know the genetic modification. However, whether these results would be reproduced at an industrial scale still needs to be addressed. Moreover, we do not know how these results would reproduce for a different population mixture (e.g., more strains growing simultaneously) or for a mutant strain in which the genetic modification performed altered its growth capability, since mutants with lower growth rates might be more difficult to trace. In our case, the parental and Δ*araR* strains show the same growth rates under control growth conditions (Fig. 1).

Although PCR sensitivity is lower than other techniques for strains identification, such as qPCR or Next Generation DNA Sequencing (NGS), it is a user-friendly, cheap, quick and specific technique that is available in all molecular biology laboratories, making it suitable for the detection of strains on a routine basis. However, we cannot discard the application of these other technologies for the traceability of strains whenever higher sensitivity for strains detection is required, although this would require more time-consuming and laborious protocols and more expensive equipment.

## Conclusions

In this study, we initiated a risk assessment analysis in which we explored the possibility of barcoding CRISPR/Cas9 genetic events by inserting a specific DNA sequence that can be used to identify genome-edited strains. For this aim, we used the industrially relevant fungal workhorse *A. niger* as a test case. We compared the efficiency of the genetic modification, the traceability and the fitness of barcoded and non-barcoded strains, and results showed that both barcoded and non-barcoded mutants can be easily and equally traceable by routine PCRs when either the specific CRISPR/Cas9 modification or the barcode sequence is known. Additionally, barcodes did not affect growth or protein production of the strains. We thus conclude that genetic barcodes are suitable tools to track CRISPR-derived GMOs without affecting the performance of the resulting strains, which can be highly advantageous, especially when dealing with pathogenic strains or in industrial applications. These results will also be particularly valuable for policy-makers and other stakeholders involved in GMO legislation, since a good understanding of the environmental risks of the CRISPR/Cas9 technology is essential to develop the rules under which it can be applied in fungi and other organisms. Further studies are needed to confirm whether these results obtained on a laboratory scale would also be reproducible on an industrial scale.

## Supplementary Information

Below is the link to the electronic supplementary material.

Supplementary file1 (PDF 300 KB)

## Declarations

### Availability of data and material

All data generated or analyzed during this study are included in this published article.
