# Supplementary material for: Genetic barcodes allow traceability of CRISPR/Cas9-derived Aspergillus niger strains without affecting their fitness
Source: Curr Genet. 2021 Mar 16;67(4):673–84. doi: 10.1007/s00294-021-01164-5 (PMC8254718; doi:10.1007/s00294-021-01164-5)
Supplement: Supplementary file 1 — Supplementary file1 (PDF 300 KB) [file 294_2021_1164_MOESM1_ESM.pdf]

Supplementary Material

Genetic barcodes allow traceability of CRISPR/Cas9-derived *Aspergillus niger* strains without affecting their fitness

Sandra Garrigues, Roland S. Kun, and Ronald P. de Vries*

Fungal Physiology, Westerdijk Fungal Biodiversity Institute & Fungal Molecular Physiology, Utrecht University, Uppsalalaan 8, 3584 CT Utrecht, The Netherlands

*Corresponding author: Ronald P. de Vries: r.devries@wi.knaw.nl

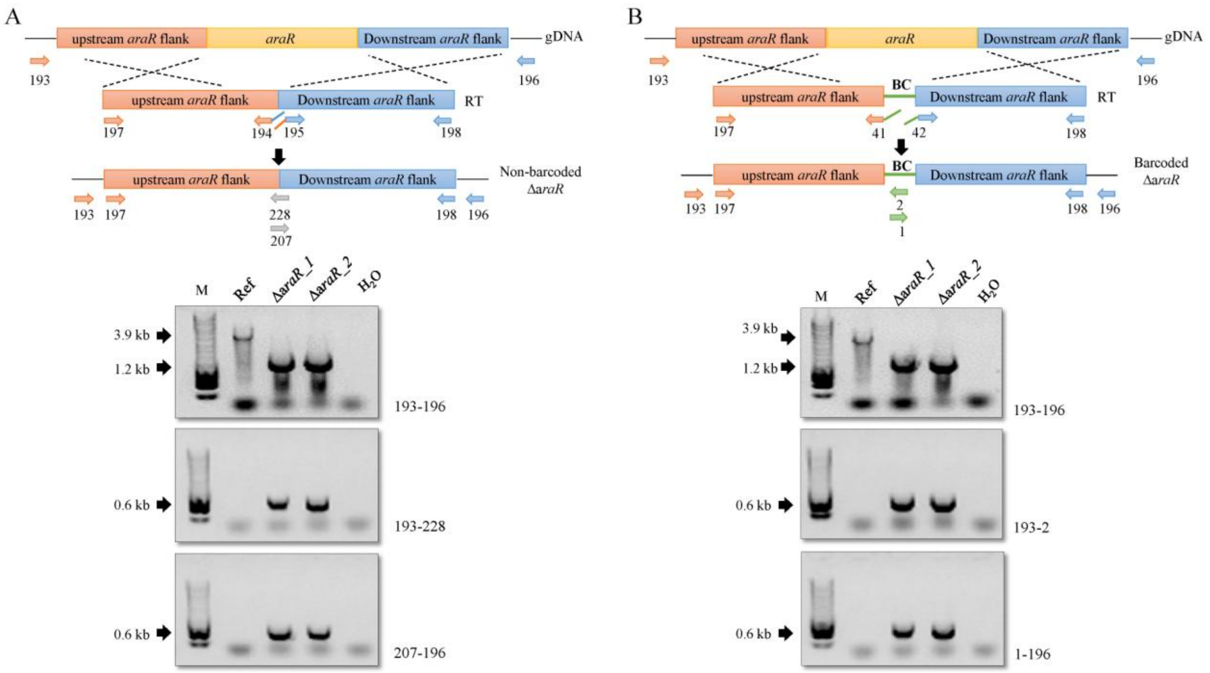

Supplementary Figure S1. Molecular confirmation of non-barcoded (A) and barcoded (B) Δ*araR* mutant strains. Graphical representation of the *araR* gene in *A. niger*, the repair templates (RT) designed to delete this gene, and the different Δ*araR* strains obtained after homologous recombination are represented on the top. Primers used to check transformants by PCR amplification are localized in the figure and represented with arrows. Results of PCR amplifications of genomic DNA from two independent non-barcoded and barcoded Δ*araR* strains using distinct primer pairs are represented on the bottom. The lower PCR bands (~0.6 and 1.2 kb) correspond to the mutants, while the higher bands (~3.9 kb) correspond to the parental strain, in which *araR* is present. Figures are not drawn to scale.

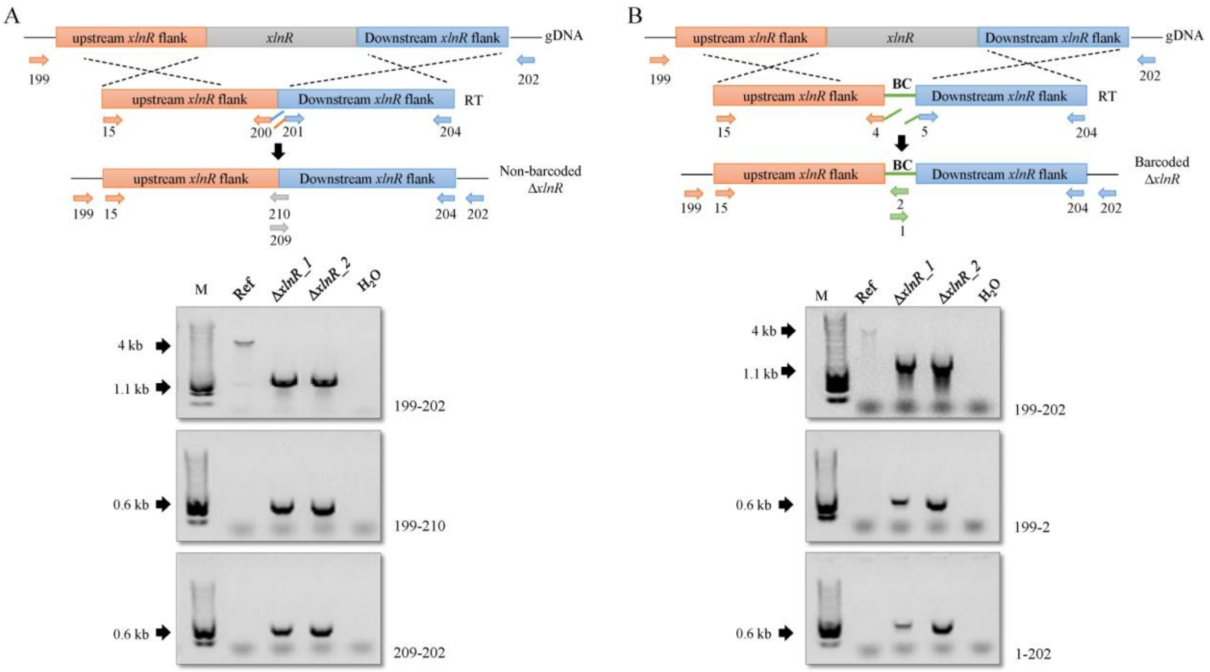

Supplementary Figure S2: Molecular confirmation of non-barcoded (A) and barcoded (B) Δ*xlnR* mutant strains. Graphical representation of the *xlnR* gene in *A. niger*, the repair templates (RT) designed to delete this gene, and the different Δ*xlnR* strains obtained after homologous recombination are represented on the top. Primers used to check transformants by PCR amplification are localized in the figure and represented with arrows. Results of PCR amplifications of genomic DNA from two independent non-barcoded and barcoded Δ*xlnR* strains using distinct primer pairs are represented on the bottom. The lower PCR bands (~0.6 and 1.1 kb) correspond to the mutants, while the higher bands (~4 kb) correspond to the parental strain, in which *xlnR* is present. Figures are not drawn to scale

A

Condition	Control DNA (ng)	Mutant DNA (ng)
1	0	10
2	0	1
3	0	0.1
4	0	0.01
5	0	0.001

B C

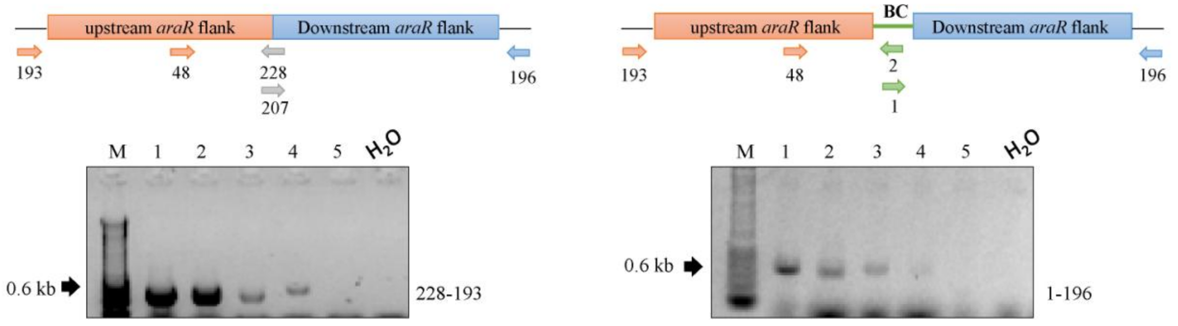

Supplementary Figure S3: Study of the traceability of the mutants without genomic DNA of the parental strain. (A) Genomic DNA amounts used for the traceability of the barcoded and non-barcoded Δ*araR* strains. Notice that no parental DNA is added. (B) Non-barcoded mutants. (C) Barcoded mutants. Numbers in each well correspond to the conditions showed in (A). Primer locations are showed on the upper schemes. Primer pair combination for each PCR reaction is shown next to the corresponding electrophoresis gel. Bands of 0.6 kb correspond to the mutants. M: molecular weight marker (HyperLadder™ 1kb, Bioline). Figures are not drawn to scale
